# Dosimetry and efficacy of a tau PET tracer [^18^F]MK-6240 in Japanese healthy elderly and patients with Alzheimer’s disease

**DOI:** 10.1007/s12149-022-01808-7

**Published:** 2022-11-21

**Authors:** Akihito Ohnishi, Go Akamatsu, Yasuhiko Ikari, Hiroyuki Nishida, Keiji Shimizu, Keiichi Matsumoto, Kazuki Aita, Masahiro Sasaki, Yasuji Yamamoto, Tomohiko Yamane, Michio Senda

**Affiliations:** 1 Department of Molecular Imaging Research, Kobe City Medical Center General Hospital, 2-1-1 Minatojima-Minamimachi, Chuo-Ku, Kobe, Hyogo 650-0047 Japan; 2 Department of Radiology, Kobe Red Cross Hospital, 1-3-1 Wakinohamakaigan-Dori, Chuo-Ku, Kobe, Hyogo 651-0073 Japan; 3 Department of Advanced Nuclear Medicine Sciences, Institute for Quantum Medical Science, National Institutes for Quantum Science and Technology (QST), 4-9-1 Anagawa, Inage-Ku, Chiba, 263-8555 Japan; 4 Department of Medical Physics and Engineering Course of Health Science, Osaka University Graduate School of Medicine, 1-7 Yamadaoka, Suita-Shi, Osaka, 565-0871 Japan; 5 Department of Radiological Technology, Kyoto College of Medical Science, 1-3 Imakita Oyamahigashi-Cho, Sonobe Nantan, Kyoto, 622-0041 Japan; 6 Department of Biosignal Pathophysiology, Kobe University Graduate School of Medicine, 1-1 Rokkodai-Cho, Nada-Ku, Kobe, 657-8501 Japan

**Keywords:** Tau PET, [^18^F]MK-6240, Dosimetry, Alzheimer’s disease, Japanese

## Abstract

**Objective:**

A new tau PET tracer [^18^F]MK-6240 has been developed; however, its dosimetry and pharmacokinetics have been published only for a European population. This study investigated the safety, radiation dosimetry, pharmacokinetics and biodistribution of [^18^F]MK-6240 in Japanese elderly subjects. Also, the pattern and extent of brain retention of [^18^F]MK-6240 in Japanese healthy elderly subjects and patients with Alzheimer’s disease (AD) were investigated. These Japanese results were compared with previous reports on non-Japanese.

**Methods:**

Three healthy elderly subjects and three AD patients were enrolled. Dynamic whole-body PET scans were acquired for up to 232 min after starting injection of [^18^F]MK-6240 (370.4 ± 27.0 MBq) for the former, while a dynamic brain scan was performed from 0 to 75 min post injection for the latter. For both groups, brain PET scans were conducted from 90 to 110 min post injection. Sequential venous blood sampling was performed to measure the radioactivity concentration in the whole blood and plasma as well as the percentages of parent [^18^F]MK-6240 and radioactive metabolites in plasma. Organ doses and effective doses were estimated using the OLINDA Ver.2 software. Standardized uptake value ratios (SUVRs) and distribution volume ratios (DVRs) by Logan reference tissue model (LRTM) were measured in eight brain regions using the cerebellar cortex as the reference. Blood tests, urine analysis, vital signs and electrocardiography were performed for safety assessments.

**Results:**

No adverse events were observed. The highest radiation doses were received by the gallbladder (257.7 ± 74.9 μGy/MBq) and the urinary bladder (127.3 ± 11.7 μGy/MBq). The effective dose was 26.8 ± 1.4 μSv/MBq. The parent form ([^18^F]MK-6240) was metabolized quickly and was less than 15% by 35 min post injection. While no obvious accumulation was found in the brain of healthy subjects, focal accumulation of [^18^F]MK-6240 was observed in the cerebral cortex of AD patients. Regional SUVRs of the focal lesions in AD patients increased gradually over time, and the difference of SUVRs between healthy subjects and AD patients became large and stable at 90 min after injection. High correlations of SUVR and DVR were observed (*p* < 0.01).

**Conclusion:**

The findings supported safety and efficacy of [^18^F]MK-6240 as a tau PET tracer for Japanese populations. Even though the number of subjects was limited, the radiation dosimetry profiles, pharmacokinetics, and biodistribution of [^18^F]MK-6240 were consistent with those for non-Japanese populations.

**Trial registration:**

Japan Pharmaceutical Information Center ID, JapicCTI-194972.

**Supplementary Information:**

The online version contains supplementary material available at 10.1007/s12149-022-01808-7.

## Introduction

Tau is the primary protein composing neurofibrillary tangles (NFT). Unlike β-amyloid deposition, post-mortem studies have shown that NFT density correlates with neurodegeneration and cognitive impairment [1–4]. Thus, PET imaging agents that bind to aggregated tau have the potential to serve as a biomarker for disease severity of Alzheimer’s disease (AD) and tauopathies, and may be useful for monitoring disease progression and therapeutic effects.

Many NFT targeted PET tracers were developed during the last several years [5, 6]. However, some of these tracers had issues regarding their specificity, sensitivity, selectivity and affinity. Recently, a new NFT targeted PET tracer [^18^F]MK-6240, which binds selectively to NFT pathology, was developed as a non-invasive imaging tool for evaluation of NFT in human brain.

The first-in-human research data of [^18^F]MK-6240 were collected in Europe [7, 8]. The data showed that [^18^F]MK-6240 had no safety concerns and was well tolerated. Because there might be ethnic differences in pharmacokinetics and radiation dosimetry profiles, it was necessary to conduct a phase I pharmacokinetic and safety study in Japanese populations as part of [^18^F]MK-6240 clinical development program in Japan. In addition, the brain accumulation in Japanese populations should be investigated to support the [^18^F]MK-6240 as a tau tracer.

Therefore, first we investigated the safety, radiation dosimetry, pharmacokinetics and whole-body biodistribution of [^18^F]MK-6240 in three Japanese elderly healthy subjects. Next, we assessed the pattern and extent of brain retention of the [^18^F]MK-6240 in the three Japanese elderly healthy subjects and three patients with AD. Finally, those results were compared with previous non-Japanese data.

## Materials and methods

### Radiopharmaceutical preparation

[^18^F]MK-6240 was radio-synthesized with the NEPTIS® Perform synthesizer and the synthesis scheme was provided by Cerveau Technology. The radio-synthesis followed the previously reported method with minor modifications [9]. Briefly, after labeling and de-protecting, a neutralized reaction mixture was transferred to the semi preparative high-performance liquid chromatography (HPLC) unit of the synthesizer. The mixture was injected onto a semi-preparative HPLC column (Phenomenex Gemini C18, 5 µm, 10 × 250 mm), and eluted with 60:40 10 mM sodium dihydrogen phosphate buffer:acetonitrile by volume, at a flow rate of 4.0 mL/min. The eluent was monitored by UV (280 nm) and radioactivity detectors connected in series. [^18^F]MK-6240 fraction was collected and transferred to the Sep-Pak t-C18 Plus Cartridge, and was washed with 15 mL of water. Trapped [^18^F]MK-6240 on the cartridge was eluted with 1 mL of ethanol and diluted with 20 mL of 0.5% sodium ascorbate in saline. The solution was passed through a 0.22 µm sterilizing filter (Merck GV33) connected to a sterile 30 mL bulk vial. The bulk product could be diluted with diluent composed of 10% ethanol and 0.5% sodium ascorbate in saline. The final formulation of the [^18^F]MK-6240 injection contains less than 10% ethanol and 0.5% sodium ascorbate. The synthesis was finished within 65 min. The total decay corrected yield was 13.7 ± 2.1%. The radio-ligand had high radiochemical purity (95%) and a molar activity of 149 ± 125 GBq/µmol at the time of injection (n = 6 batches). Other specifications for clinical use were all satisfied.

### Participants

This study was approved by the institutional review board of the Kobe City Medical Center General Hospital (19–14), and registered with the Japan Pharmaceutical Information Center (JapicCTI-194972). Written informed consent was obtained from all participants. Four healthy elderly Japanese subjects (3 males and 1 female, 60–85 years old) and 9 Japanese patients (2 males and 7 females, 71–84 years old) suspected of mild-to-moderate AD or mild cognitive impairment (MCI) underwent the screening process described below.

All persons were free of current medical and psychiatric illnesses as determined by their medical history. They were then screened based on their history, physical examination, vital signs (blood pressure, heart rate, and temperature), electrocardiography, and urine and blood tests (whole blood count, prothrombin time/activated partial thromboplastin time, albumin, alkaline phosphatase, alanine aminotransferase, aspartate aminotransferase, bicarbonate, calcium, chloride, creatinine, blood glucose, phosphorus, potassium, sodium, total bilirubin, total protein, and urea nitrogen), drug screening, and a pregnancy test for females with reproductive potential. Blood infectious disease tests (antigen screening for hepatitis B and human immunodeficiency virus (HIV), and antibody screening for syphilis, HIV, and hepatitis C), brain magnetic resonance imaging (MRI) to exclude active illnesses or abnormal changes requiring medical intervention, and chest X-ray to exclude respiratory diseases were also performed as screening tests. The persons were non-smokers or had not used nicotine-containing products for at least 3 months. Then, the persons underwent Mini-Mental State Examination (MMSE) as a neuropsychological test. The healthy subjects met the following diagnostic criteria: MMSE score of ≥ 27, no history of subjective memory or other cognitive complaints and no objective evidence of memory or cognitive impairment.

The AD patients met the diagnostic criteria for dementia of the Alzheimer’s type (mild to moderate) based on the following criteria: MMSE score of ≤ 28, Clinical Dementia Rating (CDR) score of 1 or 2, positive by qualitative analysis (visual read) of [^18^F]Flutemetamol amyloid PET imaging, being diagnosed with mild to moderate AD by an investigator as consistent with screening evaluations and meeting the criteria for AD dementia based on DSM-IV and NINCDS-ADRDA criteria.

Exclusion criteria were as follows: those who had participated in another interventional trial within 4 weeks or had undergone an radiological examination or radiotherapy with a radiation burden over 10 mSv within 12 months before the screening visit; those who had evidence of a clinically relevant neurological disorder other than Alzheimer’s disease at screening; those who had findings of an active disease requiring medical intervention on MRI scan; those who had a history or current evidence of a depressive disorder based on DSM-IV criteria; those who had a history of alcoholism or drug dependency/abuse within 2 years before screening; those who had a history of a malignant tumor, significant multiple or severe allergies; those who were positive for hepatitis B surface antigen, hepatitis C antibodies, or HIV; those who had underwent surgery, donated or lost 1 unit of blood within 4 weeks prior to the screening visit; those who had the QTc interval ≥ 470 ms for males or ≥ 480 ms for females in their electrocardiogram; those who consumed greater than 3 glasses of alcoholic beverages per day on average in the 2 weeks before injection of [^18^F]MK-6240; those who consumed greater than 6 glasses of caffeinated beverages per day on average in the 2 weeks before injection of [^18^F]MK-6240; those using cannabis or any illicit drugs; and those who would be unable to undergo MRI or PET scanning.

Based on the above screening tests including the neuropsychological tests, 3 of 4 healthy subjects (2 males and 1 female, 60–83 years old) and 3 of 9 patients (2 males and 1 female, 71–77 years old) as AD patients were enrolled in this study and proceeded to the [^18^F]MK-6240 PET scan (Table 1).

**Table 1:** Demographics of participants

Clinical diagnosis	Participant #	Sex	Age (years)	MMSE	CDR global score	MoCA-J	Injected activity (MBq)	Amyloid PET
Healthy	1	M	78	30	–	–	388	N/A
	2	F	60	30	–	–	339	N/A
	3	M	83	28	–	–	383	N/A
AD	4	M	71	15	1	10	388	Positive
	5	F	77	20	1	13	377	Positive
	6	M	76	27	1	15	386	Positive

All the mild cognitive impairment (MCI) persons were dropped at the time of screening, and none were enrolled.

### PET scans

Subjects were administered intravenously with [^18^F]MK-6240 of 377.1 ± 17.4 MBq and underwent PET scans using a Discovery 690 PET/CT scanner (GE Healthcare, Milwaukee, WI, US) [10]. The spatial resolution measured by the NEMA NU-2 procedure was 4.7 mm at a 1 cm offset position from the center of the field-of-view.

For healthy subjects, sequential whole-body PET scans were performed in the cranio-caudal direction from head to thigh. Each scan started at 1, 6, 11, 16, 25, 34, 43, 60, 150 and 200 min post injection of [^18^F]MK-6240. A total of 10 whole-body scans were performed in the following manner: 4 min (30 s/bed) × 3 times, 8 min (60 s/bed) × 3 times, 16 min (120 s/bed) × 2 times, and 32 min (240 s/bed) × 2 times (at 150–182 min and 200–232 min post injection). In addition to the whole-body scans, a brain PET scan was performed from 90 to 110 min after injection. The whole-body scans were divided into three sessions: from 1 to 77 min (first 8 scans), 150 to 182 min (9th scan) and 200 to 232 min (10th scan), with short breaks between the brain scan and the 9th scan, and between the 9th scan and the 10th scan, respectively. A low-dose CT for attenuation correction was performed before the first PET scan at each session. Sequential venous blood samplings of about 6 mL were performed before injection and 2, 6, 16, 35, 60, 90 min after [^18^F]MK-6240 injection for measurement of whole-blood and plasma radioactivity concentrations. Blood samples and vital signs were obtained before the [^18^F]MK-6240 injection and 240 min post injection. Electrocardiograms were recorded before the injection and 10, 120 and 240 min post injection. Urine was collected before the injection and at about 120 and 240 min post injection.

For AD patients, dynamic brain imaging from 0 to 75 min post injection was performed, followed by a static brain scan from 90 to 110 min after injection. Sequential venous blood sampling of about 6 mL was performed before injection and 2, 6, 16, 35, 60, 90 min after [^18^F]MK-6240 injection for measurement of the whole-blood and plasma radioactivity concentrations. Blood and urine samples, and vital signs were obtained before [^18^F]MK-6240 injection and 110 min post injection. Electrocardiograms were recorded before injection and 10, 45 and 110 min post injection.

### Whole-body PET data analysis and dosimetry

Whole-body PET data were reconstructed using an ordered-subset expectation–maximization (OSEM) algorithm (3 iterations and 8 subsets) with time-of-flight (VUE Point FX). A Gaussian filter with 4 mm FWHM was applied to the reconstructed PET images. The matrix size was a 192 × 192 with a pixel size of 3.12 mm. The slice thickness was 3.27 mm.

Image analysis was performed with the PMOD Ver.3.4 (PMOD Technologies, Switzerland). For whole-body PET data, regions of interest (ROIs) were placed over major organs (brain, lungs, heart wall, liver, spleen, gallbladder, kidneys, urinary bladder, stomach, small intestine, upper large intestine, lower large intestine, and red bone marrow) on PET/CT images. The activity in each source organ and the remainder were divided by the injection activity to obtain the uptake as a percent of the injected activity (%ID). The residence time in each source organ was calculated from the percent injection activity of each source organ and the time course information by fitting a bi-exponential curve using OLINDA Ver.2 software (Vanderbilt University) [11]. Urine was collected before injection and 110 min and 240 min post injection. Voided urine radioactivity was measured using a gamma counter (Auto Well Gamma System, ARC-370, Aloka Co.) and subtracted from the activity of the remainder. Dosimetry (effective dose) was calculated by the OLINDA Ver.2 using the adult male phantom. In addition to the OLINDA Ver.2, we calculated the dosimetry using the OLINDA Ver.1 for comparison to the European dosimetry data on [^18^F]MK-6240. The same %ID and the same residence time in source organs were used both for the OLINDA Ver.1 and the OLINDA Ver.2, whenever applicable.

### Brain PET data analysis

For brain data, PET images were reconstructed using the OSEM (VUE Point HD) with 4 iterations and 16 subsets. A Gaussian filter with 5 mm FWHM was applied to the PET images. The matrix size was 128 × 128, the pixel size was 2.73 mm, and the slice thickness was 3.27 mm. The image spatial resolution was 6 mm as assessed by visual similarity to digital phantom images applied with Gaussian filter of various FWHMs [12, 13].

The tracer uptake in each brain region was quantitatively measured with the PNEURO tool implemented in the PMOD software. The PET images were co-registered to the individual 3D T1-weighted MRI based on the normalized mutual information method. The co-registered PET images were spatially normalized to the standard Montreal Neurological Institute (MNI) T1 atlas with the same transformation parameters of MRI-based normalization. For the 0–75 min dynamic PET data, which were measured only for the patient group, the last 15-min image (60–75 min after injection) was used for image registration. The automatic anatomical-labeling (AAL) ROI template was applied to PET images in a native space using the inverse transformation parameters of the co-registration and spatial normalization. The inversely transformed ROI was masked with the gray matter segmented by individual MRI-based parcellation. Standardized uptake values (SUV) were measured in the following brain regions: frontal cortex, mesial temporal cortex, lateral temporal cortex, parietal cortex, occipital cortex, anterior cingulate, posterior cingulate, hippocampus and parahippocampus. The standardized uptake value ratio (SUVR) in each region was also calculated using the cerebellar cortex as a reference.

For brain kinetic analysis, the Logan reference tissue model (LRTM) was used to calculate the distribution volume ratio (DVR_LRTM_) of each brain region using the PKIN tool [14]. The t* (equilibration start time) and k’_2_ were set to 15 min and 0.144 min^−1^, respectively [7]. To confirm the SUVR as an appropriate metric for [^18^F]MK-6240 specific binding, the correlation between SUVR and DVR_LRTM_ was investigated.

### Analysis of metabolites in blood samples

Metabolite analysis was performed by the literature method with minor modifications [15]. Sequential venous blood samplings (about 6 mL) were performed before injection and 2, 6, 16, 35, 60, 90 min after [^18^F]MK-6240 injection to measure whole-blood and plasma radioactivity concentrations with a gamma counter (radio-detection linearity range: 10–25,000 Bq, Auto Well Gamma System, ARC-400, Aloka Co.) and to measure parent [^18^F]MK-6240 and its radioactive metabolites in the de-proteinated plasma by HPLC. HPLC system conditions were the following: HPLC module, SHIMADZU AD-10 series (pump, UV detector, column oven, controller) (SHIMADZU, Co., Ltd. Japan); radiation detector, US-2000 (1 inch × 1 inch NaI(Tl), Universal Giken Co., Ltd., Japan); radio-detection limit, 500 Bq/Peak; column, Luna C18, 10 µm, 10 × 250 mm (Phenomenex Inc., CA, US); elution, methanol: water + 0.2% (v/v) triethylamine = 75:25; flow rate, 4 mL/min; UV wavelength, 254 nm.

### Statistical analysis

Because of the small number of subjects, descriptive values were presented without any statistical tests between healthy subjects and AD patients.

## Results

### Safety measures

No clinically meaningful trends were observed in the results from physical examination, measurement of vital signs, electrocardiograms, urine tests, and blood tests until the day after injection of [^18^F]MK-6240.

### Pharmacokinetics and metabolites in plasma

**Fig. 1:**
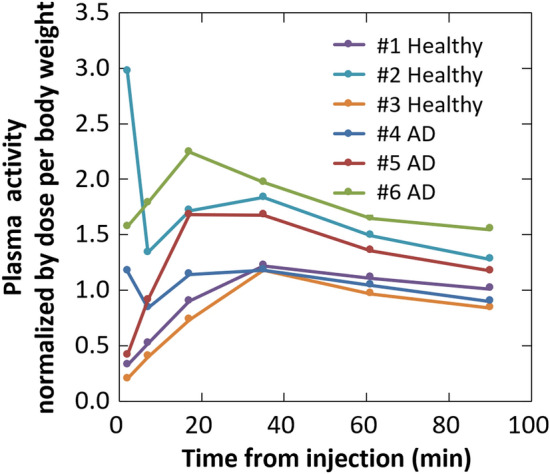
Plasma time–activity curve of each participant normalized by injected activity per body weight. The gradual increase in the plasma activity should be noted

Figure 1 shows plasma activity curve of each participant. Only one healthy subject (#2) showed the typical high peak at 2 min post injection with a rapid decrease that is often observed immediately after injection of many PET tracers. For all subjects, the plasma radioactivity concentrations gradually increased and peaked at a low value at around 16 min or 35 min after injection of [^18^F]MK-6240 and then decreased slowly. Because the ratio of whole blood to plasma was almost the same over time (healthy, 0.64 ± 0.03; AD patients, 0.64 ± 0.01), whole blood radioactivity concentrations also showed the same trends as those of the plasma.

**Fig. 2:**
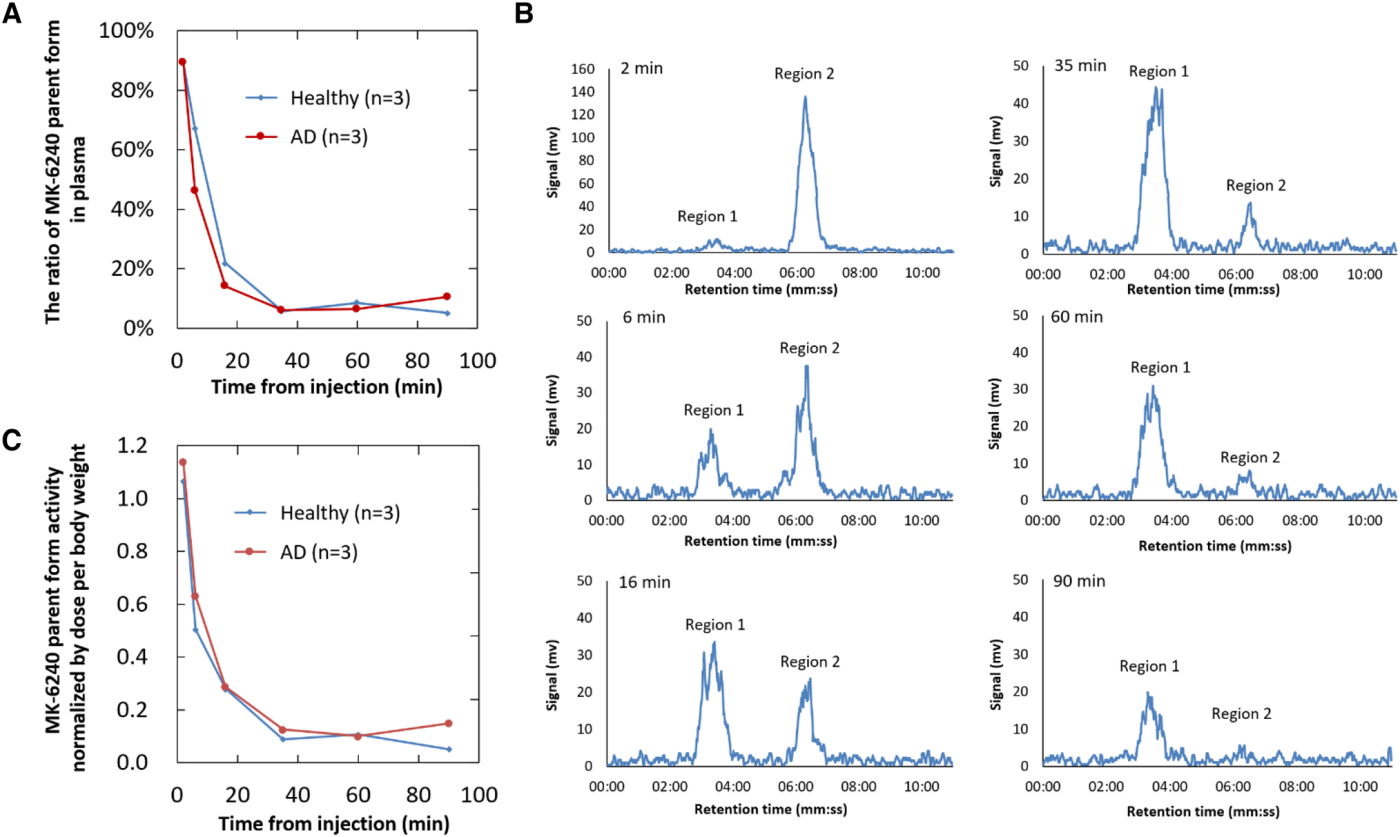
**A** Radio-HPLC results. Ratios of [^18^F]MK-6240 parent form in the total plasma radioactivity averaged for healthy subjects and AD patients (mean ± SD). **B** Radio-HPLC charts of the plasma sample obtained from a representative subject (healthy subject 2) at 2, 6, 16, 35, 60 and 90 min post injection of [^18^F]MK-6240. The vertical axis shows “Radioactivity detector response (mV)” while the horizontal axis show “Retention time (min)”. Region 2 corresponds to the [^18^F]MK-6240 parent form and region 1 corresponds to a hydrophilic metabolite. All participants showed similar trends. **C** Plasma concentration of [^18^F]MK-6240 parent form averaged for each group normalized by injected activity per body weight

In the plasma metabolite analysis with HPLC, the parent form ([^18^F]MK-6240) was metabolized quickly from the plasma and the fraction of the parent form in the circulating radioactivity was less than 15% [average ± SD; 5.7 ± 6.5% (healthy subjects) and 6.1 ± 2.7% (AD patients)] by 35 min after injection in all healthy subjects and AD patients (Fig. 2A). By that time, more than 87% [average ± SD; 94.8 ± 7.2% (healthy subjects) and 93.9 ± 2.7% (AD patients)] of ^18^F was replaced by a hydrophilic metabolite, and the parent form had almost disappeared at 60 min post injection in all healthy subjects and AD patients (Fig. 2B).

The plasma time–activity curve (TAC) of the parent form ([^18^F]MK-6240) normalized by the injection activity per body weight presented no apparent difference in clearance speed between healthy subjects and AD patients (Fig. 2C).

### Biodistribution and radiation dosimetry

**Fig. 3:**
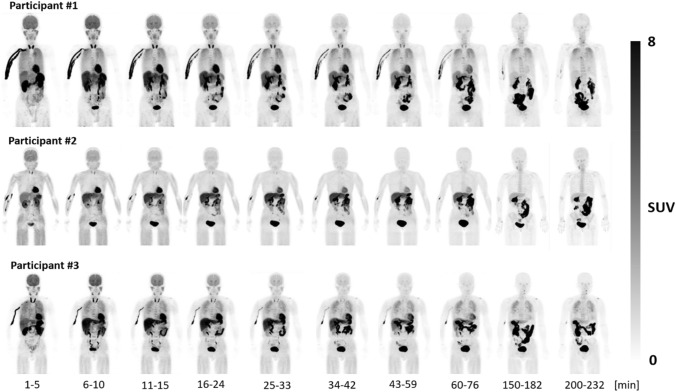
Maximum intensity projection (MIP) whole-body PET images of three healthy subjects obtained with sequential whole-body scans after injection of [^18^F]MK-6240. High radioactivity uptakes were observed mainly in organs of metabolism and excretion including liver, gallbladder, intestines, and urinary bladder

In the whole-body images in healthy subjects, uptakes of organs, such as brain, salivary glands, thyroid gland, heart, vertebral bone, liver, and kidney, and adsorption to the injected veins, were visible at the early phase. In two subjects (#1, #3), lung accumulations were observed at the early phase. In the other subject (#2), lung accumulations were not observed at the early phase, but mild muscle accumulations were found instead. Although uptake in the lungs, heart, liver, and thyroid gland, and adsorption to the injected veins remained until the late phase, the radioactivity was slowly washed out. The major excretory pathways of radioactivity were the urinary bladder via kidney and the intestine via the biliary system (Fig. 3).

**Fig. 4:**
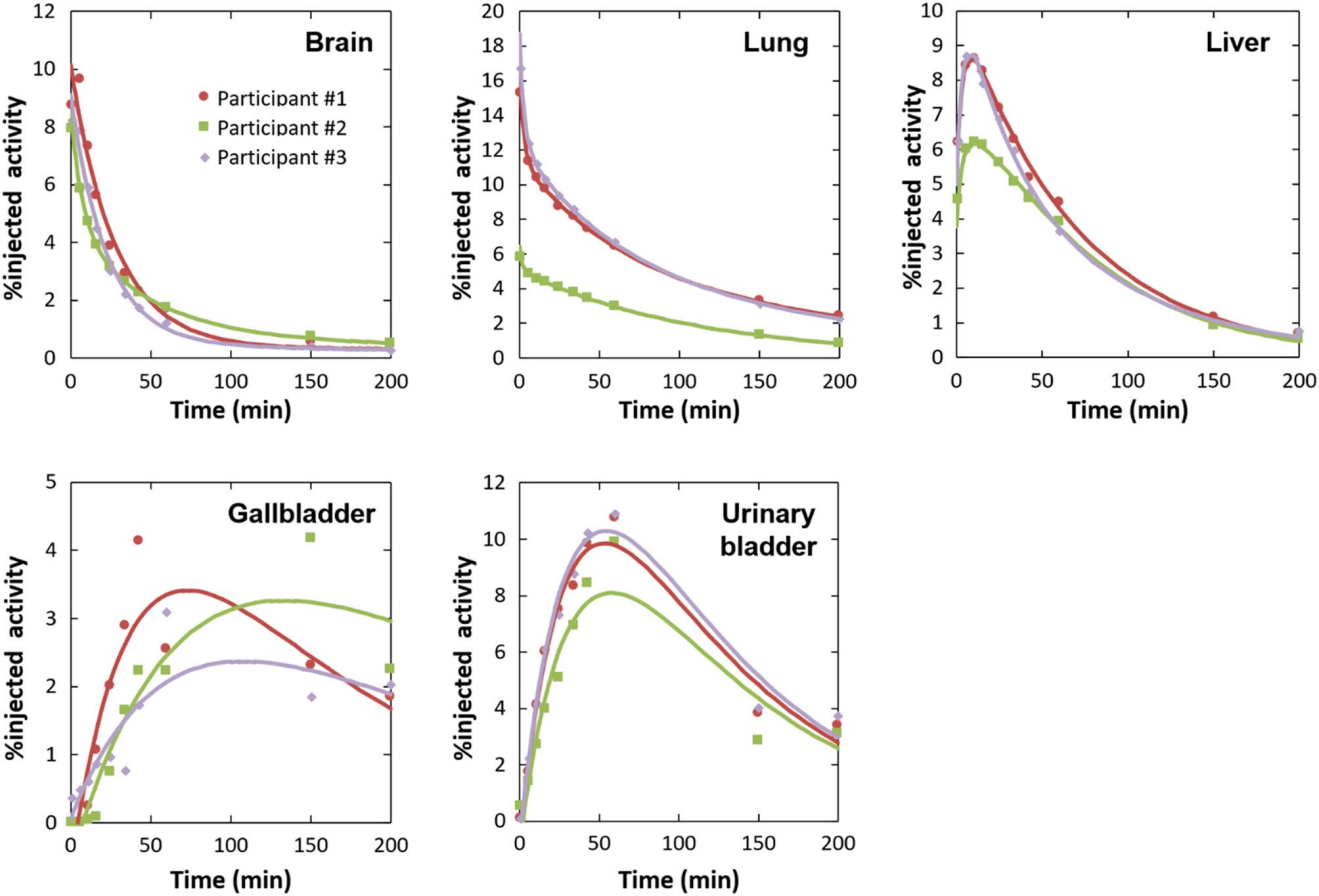
Time–activity curves of five source organs (brain, lung, liver, gallbladder, and urinary bladder) after injection of [^18^F]MK-6240 for three healthy subjects. The whole organ radioactivity was expressed as %injected activity (%ID) that are fitted by exponential curves (solid lines)

The organ time–activity curves for brain, lung, liver, gallbladder, and urinary bladder are shown in Fig. 4. The highest mean initial uptakes of radioactivity were found in the lungs (12.6%) and the brain (8.3%). They were followed by a rapid clearance over the duration of the scan.

Higher retentions of radioactivity were found in gallbladder, small intestine, and urinary bladder. They were due to hepatobiliary and renal clearance of the tracer. The organs with the highest mean absorbed dose (mean ± SD) were the gallbladder (257.7 ± 74.9 μGy/MBq), the urinary bladder (127.3 ± 11.7 μGy/MBq), the left colon (74.9 ± 32.5 μGy/MBq), the right colon (74.7 ± 34.2 μGy/MBq), and the small intestine (54.1 ± 27.1 μGy/MBq). The mean effective dose (mean ± SD) was 26.8 ± 1.4 μSv/MBq (Table 2). Supplemental Table 1 shows the dosimetry data calculated by OLINDA Ver.1. Effective dose for a single injection of 185 MBq (5 mCi) of [^18^F]MK-6240, which is the standard injection activity of the PET drug, was expected to be 5.0 mSv.

**Table 2:** Radiation dosimetry estimates (OLINDA ver. 2.2) for [^18^F]MK-6240: Organ doses and effective dose (written in **bold**) estimated from three elderly healthy subjects

Target organs	Organ dose (µGy/MBq)
Adrenals	16.7 ± 0.5
Brain	17.9 ± 6.9
Esophagus	11.8 ± 1.3
Eyes	6.5 ± 0.6
Gallbladder wall	257.7 ± 74.9
Left colon	74.9 ± 34.2
Small intestine	54.1 ± 27.1
Stomach wall	16.4 ± 1.3
Right colon	74.7 ± 32.5
Rectum	14.9 ± 0.9
Heart wall	29.7 ± 6.3
Kidneys	27.8 ± 1.5
Liver	24.7 ± 1.0
Lungs	33.3 ± 12.8
Pancreas	17.0 ± 1.3
Prostate	16.4 ± 0.8
Salivary glands	7.4 ± 0.5
Red marrow	13.7 ± 0.5
Osteogenic cells	10.9 ± 0.4
Spleen	13.3 ± 2.9
Testes	7.6 ± 0.2
Thymus	10.5 ± 1.2
Thyroid	8.5 ± 0.7
Urinary bladder wall	127.3 ± 11.7
Total body	10.3 ± 0.4
**Effective dose (µSv/MBq)**	**26.8 ± 1.4**

Data are mean ± SD

### Brain distributions and SUVR analysis

**Fig. 5:**
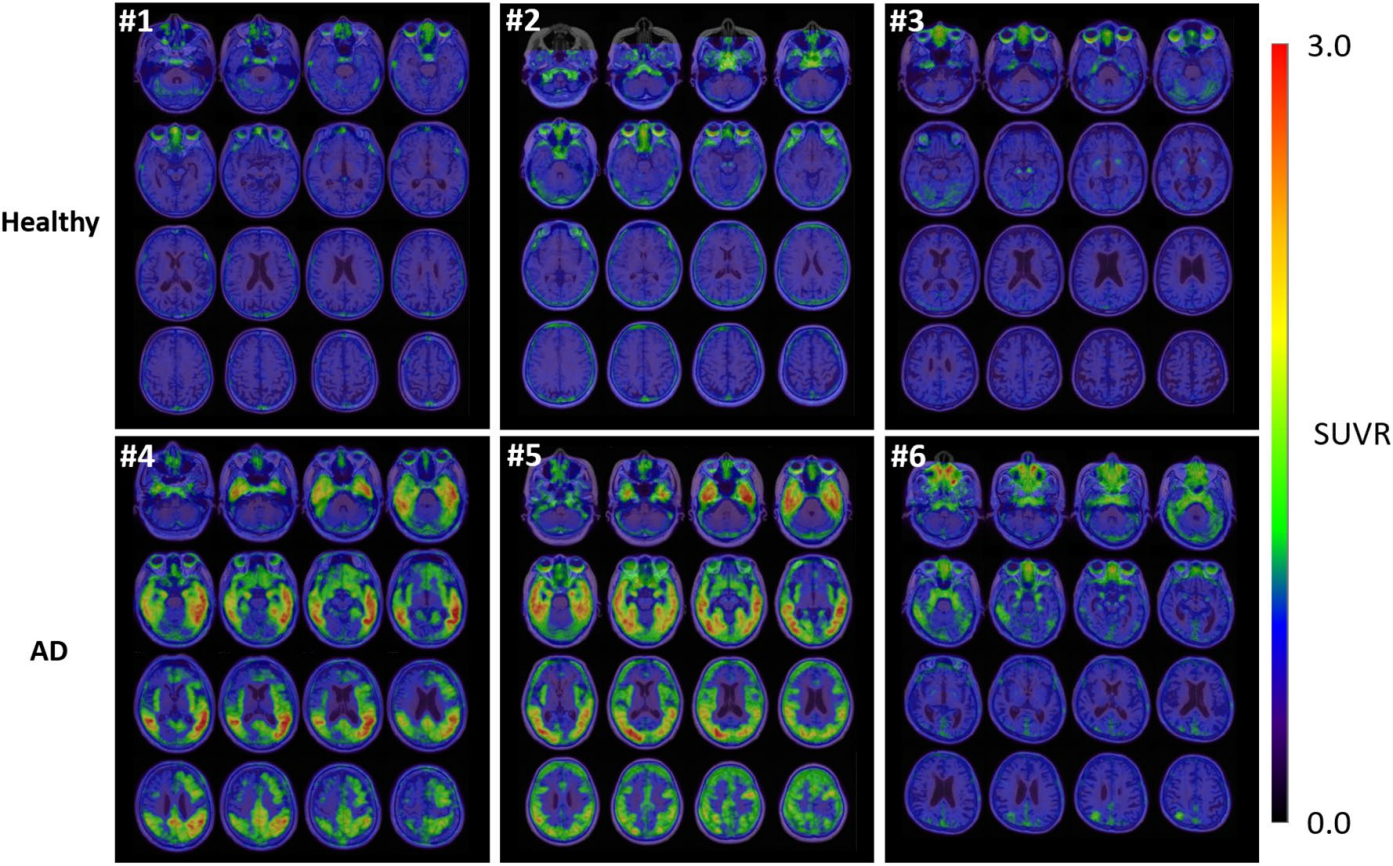
Brain PET images acquired from 90 to 110 min post injection of [^18^F]MK-6240 in healthy subjects and AD patients. No obvious accumulation was found in the entire brain in healthy subjects (#1–3). Accumulations in temporal lobe, occipital lobe and parietal lobe and mild accumulation in frontal lobe were found in two patients (#4 and #5). The focal mild uptake in the temporal lobe was found in the third patient (#6). Subject information is listed in Table 1

In brain images from 90 to 110 min after [^18^F]MK-6240 injection, no clear accumulation was found in the entire brain of healthy subjects (Fig. 5). On the other hand, focal mild uptake of [^18^F]MK-6240 was found in the temporal lobe of patient #6, and accumulations were found in the temporal, occipital, parietal and frontal lobes of patients #4 and #5. In patient #4, clear accumulations in the posterior cingulate and precuneus were also observed, and mild accumulations in the posterior cingulate and precuneus were observed in the other patients. Off-target uptakes were found in the basal cavity, paranasal sinus, bone marrow, meninges, and retina.

**Fig. 6:**
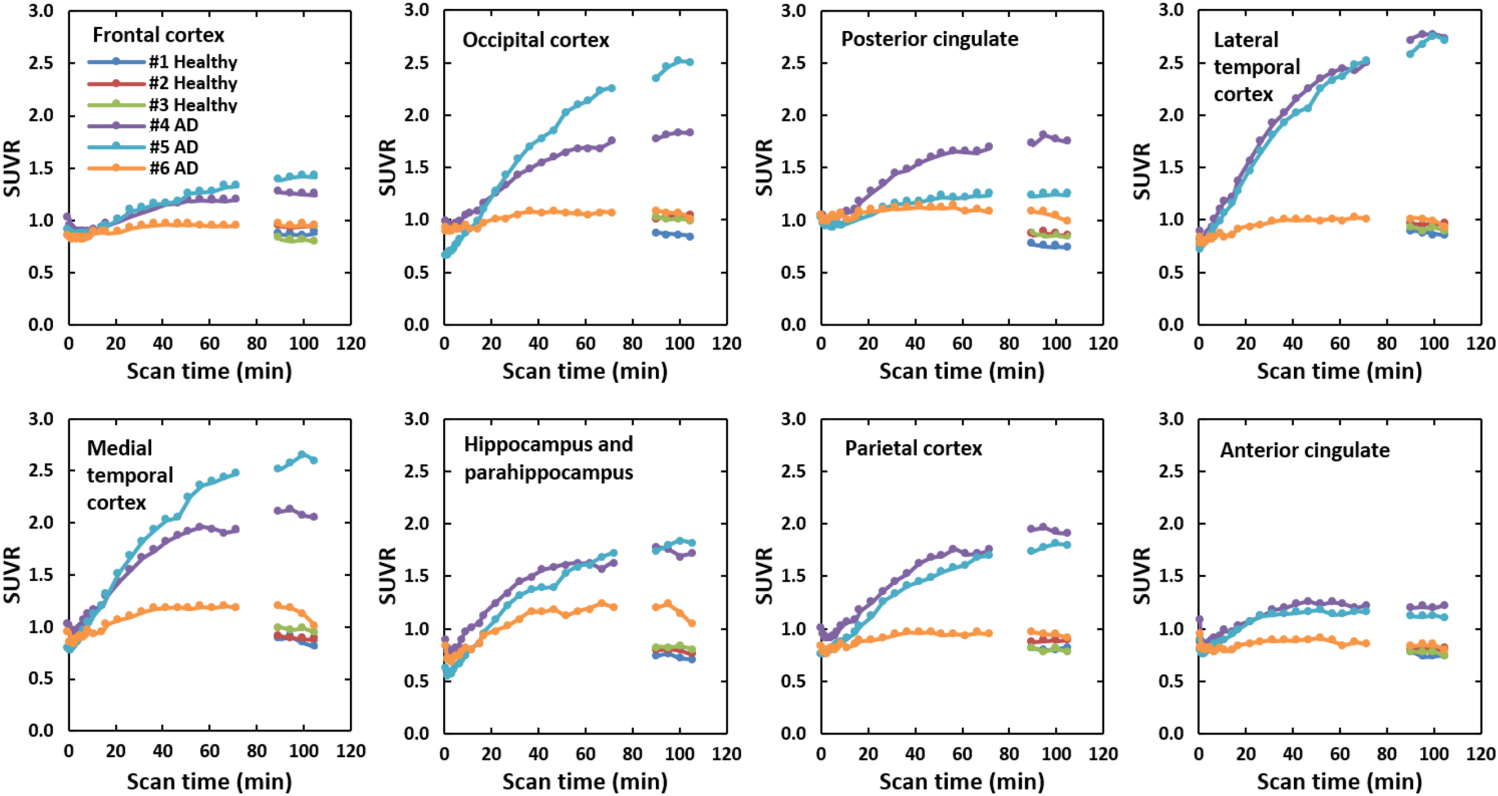
Time course of SUVR in each brain region: frontal cortex, occipital cortex, posterior cingulate, lateral temporal cortex, medial temporal cortex, hippocampus and parahippocampus, parietal cortex and anterior cingulate. SUVRs for AD patients increased gradually over time. In the 90 to 110 min period after injection, large differences in SUVRs for these brain regions were observed between healthy subjects and AD patients

In most brain regions, SUVRs were stable at 90 min after injection. In those regions of AD patients that presented an abnormal uptake in the 90–110 min brain images, SUVRs increased gradually over time and were higher than those of healthy subjects between 90 and 110 min (Fig. 6).

### Brain SUVR-DVR correlation

**Fig. 7:**
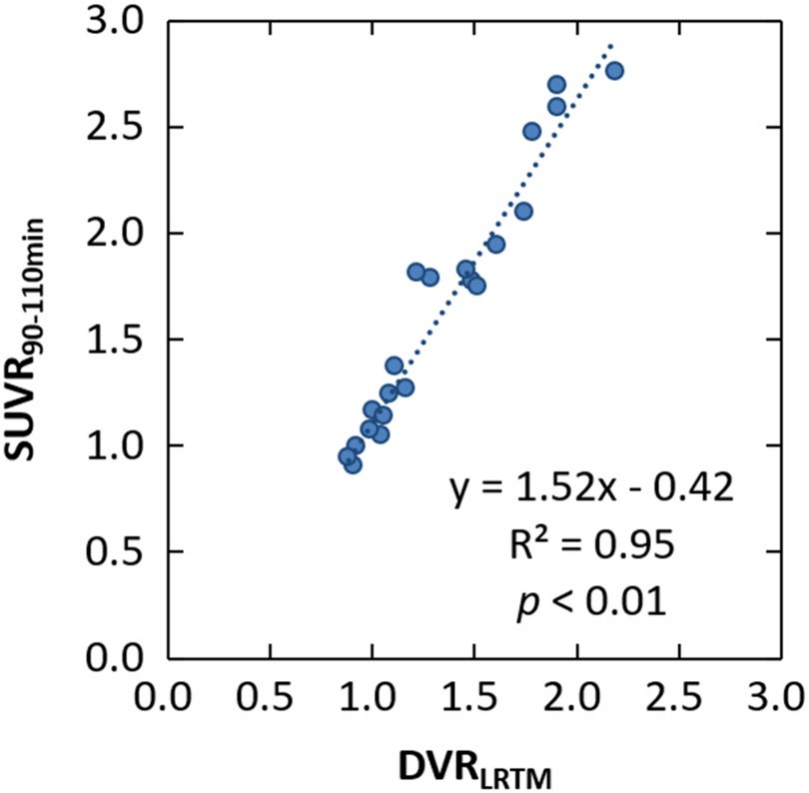
Correlations between the SUVR_90-110 min_ and DVR_LRTM_ in three AD patients. Measured brain regions were frontal cortex, mesial temporal cortex, lateral temporal cortex, parietal cortex, occipital cortex, anterior cingulate, posterior cingulate, hippocampus and parahippocampus

A correlation between SUVR and DVR_LRTM_ for various regions of the three AD patients is shown in Fig. 7. SUVR and DVR_LRTM_ showed a linear correlation with the slope of 1.52 (R^2^ = 0.95, *p* < 0.01). Supplemental Fig. 2 shows the LRTM plot for the temporal regions of each AD subject, and the linearity allowed calculation of DVR_LRTM_ from the slope.

## Discussion

### Safety, biodistribution and radiation dosimetry

We presented the results of the safety profile, whole-body biodistribution and radiation dosimetry for [^18^F]MK-6240 in healthy elderly Japanese subjects. The results indicated that [^18^F]MK-6240 is a safe radiopharmaceutical.

The effective dose of [^18^F]MK-6240 was 26.8 ± 1.4 μSv/MBq with OLINDA Ver.2 and was 26.4 ± 1.2 μSv/MBq with OLINDA Ver.1, which was both comparable to the 29.4 ± 0.6 μSv/MBq reported by Koole et al. [8] as evaluated by OLINDA Ver.1.1 for non-Japanese subjects. It was also similar to the effective dose of other ^18^F-labeled PET tracers such as [^18^F]AV-1451 ([^18^F]flortaucipir), another commonly used tau PET tracer of which the effective dose was estimated to be 22.5 μSv/MBq [16]. When 185 MBq of [^18^F]MK-6240 is administered, the total effective dose is estimated to be 5.0 mSv. Thus, the radiation exposure risk is within an acceptable range.

We used the adult male phantom implemented in the OLINDA software. The phantom was generated based on data on Western Europeans and North Americans [17]. Therefore, the phantom size including the target organ masses was different from that of the Japanese. This is one of the limitations of this study.

The radiation doses to organs of excretion (bladder, gallbladder, small intestine, colon) were relatively high. Koole et al. [8] also showed that radiation doses to organs of excretion (bladder, 128 µGy; gallbladder, 202 µGy; small intestine, 116 µGy; lower large intestine wall, 46.4 µGy; upper large intestine wall, 128 µGy) were higher than those of other organs. The trends for high radiation dose organs were the same as those of the present study. In the early phase of whole-body images, the radioactivity accumulated in highly perfused organs, such as the brain, myocardium, liver, and kidneys. For two healthy subjects, uptakes in the lungs were observed in the early phase. Uptakes in the thyroid glands and salivary glands were also observed. [^18^F]MK-6240 broadly distributed during single perfusion. Radioactivity in these organs was gradually washed out. For the same two healthy subjects, the lung uptake remained until the late phase. As for the hepatobiliary excretion, the bile duct, gallbladder, and intestinal tract were visualized as the liver’s uptake was gradually washed out. Similarly, as the kidneys’ cortical uptake was gradually washed out, the renal pelvis, ureter, and bladder were visualized. These indicated that the radiopharmaceutical is excreted from the liver and kidneys. Time–activity curves of brain, liver, gallbladder, and urinary bladder indicated the same trend as those of previously reported [8].

The radioactivity of the blood vessels of the upper limb on the administration side was conspicuous, which means [^18^F]MK-6240 would be adsorbed to the vascular wall. The degree of adsorption was different among the three subjects. Koole et al. [8] did not mention the lung uptake or vascular adsorption, and such uptakes were not found in the figures of their representative case. The vascular adsorption might be different between Japanese and non-Japanese persons or might be derived from the differences in the synthesis and formulation of [^18^F]MK-6240, although the quality standard of the PET drug used here is the same as that of the non-Japanese studies. The lung dose in the present study (33.3 ± 12.8 μGy/MBq) was higher than that of the non-Japanese study (19.7 ± 3.6 μGy/MBq) [8], possibly due to the lung uptake in two subjects The lung uptake might have individual variations rather than an ethnic difference because it was not observed in one healthy subject of the present study. Further investigations are needed to clarify the reasons and implications for lung uptakes and vascular adsorptions.

The safety tests, radiation dosimetry and drug metabolism data obtained in the present study allow clinical physicians and researchers to proceed to further studies examining patients with AD and MCI using [^18^F]MK-6240.

### Pharmacokinetics and metabolites in plasma

Results of the HPLC analysis indicated that plasma [^18^F]MK-6240 is rapidly metabolized to a hydrophilic metabolite form (Fig. 2).

The parent form was less than 15% at 35 min post injection. At that time, more than 87% of ^18^F was replaced by the hydrophilic form. These results were consistent between healthy subjects and AD patients, and were also consistent with previous reports [7, 8, 18]. These earlier results indicated that the parent form of [^18^F]MK-6240 cleared quickly to 10 ± 5% [7], less than 30% [8] by 30 min after injection, and approximately 30% by 10 min after injection [18]. In these non-Japanese data, the metabolite was one hydrophilic form. Guehl, et al. [19] also indicated a rapid metabolism of [^18^F]MK-6240, which cleared to 15% at 15 min after injection, but they detected two hydrophilic forms as the metabolites of [^18^F]MK-6240. This difference in the number of metabolite forms would be attributed to the difference of the HPLC measurement setting. This is not a clinically significant issue, however, because the metabolites were all hydrophilic forms and would not enter the brain.

The shape of the plasma time–activity curves in this study differed from that in previous reports [7, 19]. Lohith, et al. [7] showed that [^18^F]MK-6240 concentration in plasma peaked at 30–40 s post injection and then rapidly decreased. Guehl, et al. [19] showed [^18^F]MK-6240 concentrations in whole blood (whole blood to plasma ratio was 0.66 ± 0.01) peaked rapidly post injection and then rapidly decreased. In the present study, however, the plasma radioactivity gradually increased up to about 17 min or 35 min post injection and then decreased. Because uptakes in the vascular walls and lungs were observed in the early phase, their release might have caused the gradual increase in the plasma. This association is supported by a supplemental analysis that compared the increase of %injected activity in plasma with the decrease in %injected activities of the vessel retention and lung accumulation. As shown in Supplemental Fig. 1, the plasma increase was well balanced with the sum of the decrease in vessels and lungs, the differences being within 2% of %injected activity. On the other hand, the [^18^F]MK-6240 concentrations in plasma from 35 min after injection were consistent with those in previous non-Japanese studies. Although the plasma radioactivity gradually increased until about 30 min post injection, the [^18^F]MK-6240 parent form radioactivity which corresponds to the input to the brain, monotonically decreased after injection. These results suggested that the gradual increase in plasma would not have a major effect on the brain pharmacokinetics except in the early phase.

### Brain distributions and SUVR analysis

Off-target bindings were observed in the basal cavity, paranasal sinus, bone marrow, meninges and retina. These off-target regions were the same as those of the previous non-Japanese data [19, 20]. While no obvious accumulation of [^18^F]MK-6240 was found in the brain of healthy subjects, cortical uptakes of [^18^F]MK-6240 were observed in the AD patients. High uptakes were observed in the temporal cortex including hippocampus and parahippocampus of all AD patients, and in the parietal, posterior cingulate and occipital lobe in two of the three patients in this study. Thus, the brain accumulation pattern in the patients was consistent with the accumulation pattern associated with AD NFT deposition [7, 18–20].

The difference of regional SUVRs between healthy subjects and AD patients became larger over time and became stable and prominent between 90 and 110 min in most regions. Therefore, the period of 90 min post injection of [^18^F]MK-6240 would be appropriate for differentiating AD patients from healthy subjects, as well as for semiquantitative evaluation with SUVR. This is consistent with the previous non-Japanese studies which also used SUVRs at 90–110 min or 90–120 min post injection as a semiquantitative indicator [18].

Tau pathology in AD has been shown to progress in a typical pattern of spread, called Braak staging [21], among brain regions along nerve fiber connections. The earliest area of AD-related NFT deposition is considered to be the medial temporal cortex, and then NFT extends to the lateral temporal, occipital, parietal and finally frontal cortices.

In this study, one AD patient with a relatively low degree of cognitive decline based on the clinical cognition scores showed an accumulation in a limited part of the temporal lobe. The other two AD patients with more advanced cognitive decline showed a more extensive cerebral accumulation. The results were consistent with previous non-Japanese data, which showed that the range of accumulation of [^18^F]MK-6240 tended to spread as MMSE became lower, and the results supported the efficacy of [^18^F]MK-6240 as a tau PET tracer for the Japanese population.

### Brain SUVR-DVR correlation

A high linear correlation between SUVR_90-110_ and DVR_LRTM_ was observed with the slope of 1.52 (Fig. 7 and Supplemental Fig. 2) in AD patients. This result supported reversible kinetics of [^18^F]MK-6240 as demonstrated by previous studies on non-Japanese subjects. Pascoal, et al. [18] showed that DVR_LRTM_ was highly correlated with the SUVR_90-110_ (slope: 1.11). Lohith, et al. [7] also showed DVR_LRTM_ was well correlated with SUVR calculated from 60 to 90 min post injection using the cerebellar cortex as a reference (slope: 0.9–1.51). The magnitude of DVR_LRTM_ values in the regions of abnormal uptake were also consistent with those of previous non-Japanese studies. Therefore, there would be no apparent difference in brain pharmacodynamics between Japanese and non-Japanese. Although the slope of 1.52 was somewhat higher than that for non-Japanese studies, it is not clear whether it is an ethnic difference because measured DVR and SUVR are highly variable [22]. In addition, Zhou, et al. [23] reported that the LRTM tended to underestimate DVR and that SUVR tended to overestimate DVR. Therefore, although NFT deposition may be quantitatively evaluated with SUVR in [^18^F]MK-6240 PET, further verification on more subjects is required to practically use the SUVR as a quantitative marker.

## Conclusion

The safety, biodistribution and radiation dosimetry profiles in Japanese healthy elderly following the administration of [^18^F]MK-6240 have been presented. The effective dose of [^18^F]MK-6240 was 26.8 ± 1.4 μSv/MBq, which was comparable to the value of the previous non-Japanese study of [^18^F]MK-6240 and within the typical range of other ^18^F-labeled tracers. In addition, the pattern and extent of brain [^18^F]MK-6240 retention were different between healthy elderly subjects and patients with mild to moderate AD. This supported the efficacy of [^18^F]MK-6240 as a tau tracer for AD. Based on brain regional SUVR curves, an appropriate measurement period would be 90 min post injection of [^18^F]MK-6240 to differentiate healthy subjects and AD patients. The NFT deposition can be quantified by SUVR, of which reliability was supported by the linear correlation to DVR_LRTM_. Overall, the results of this study were consistent with those of the study for non-Japanese people.

## Supplementary Information

Below is the link to the electronic supplementary material.

Supplementary file1 (DOCX 194 KB)
