# Supplementary material for: Dosimetry and efficacy of a tau PET tracer [18F]MK-6240 in Japanese healthy elderly and patients with Alzheimer’s disease
Source: Ann Nucl Med. 2022 Nov 21;37(2):108–20. doi: 10.1007/s12149-022-01808-7 (PMC9902412; doi:10.1007/s12149-022-01808-7)
Supplement: Supplementary file 1 — Supplementary file1 (DOCX 194 KB) [file 12149_2022_1808_MOESM1_ESM.docx]

**Supplemental Figure 1**

Changes in the total activity (per injected activity) in the plasma (increase), vessels on the injection side (decrease) and the lungs (decrease) from 6 min to 16 min and from 16 min to 35 min for each healthy elderly subject. Circulating blood volume was assumed to be 1/13 of body weight and circulating plasma volume was calculated as 55% of the circulating blood volume.


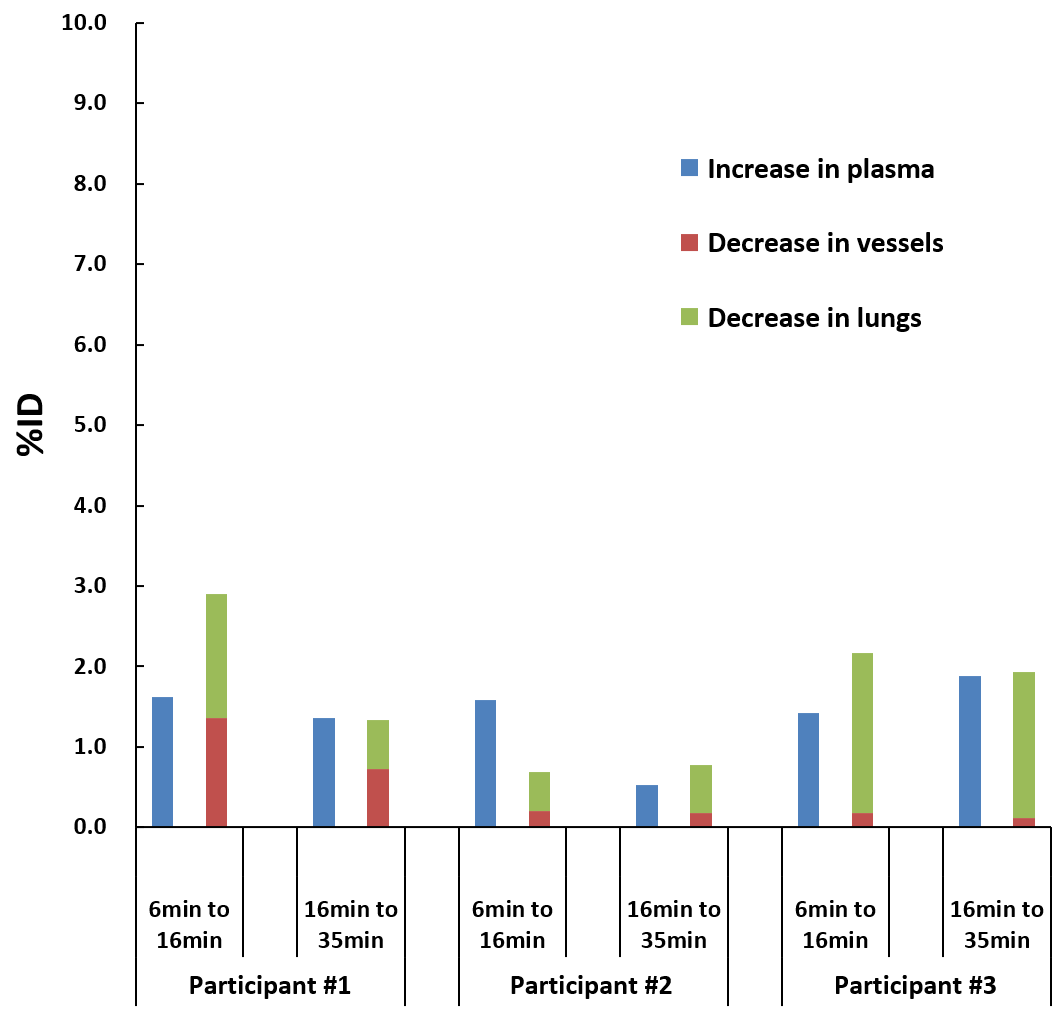


**Supplemental Figure 2**

Logan reference tissue model (LRTM) plot and linear regressions for medial temporal cortex and hippocampus and parahippocampus of each AD patient. Cref is activity concentration in the reference tissue (cerebellar cortex), Ct is activity concentration in the target region, and k’2 is average effective tissue-to-plasma efflux constant.


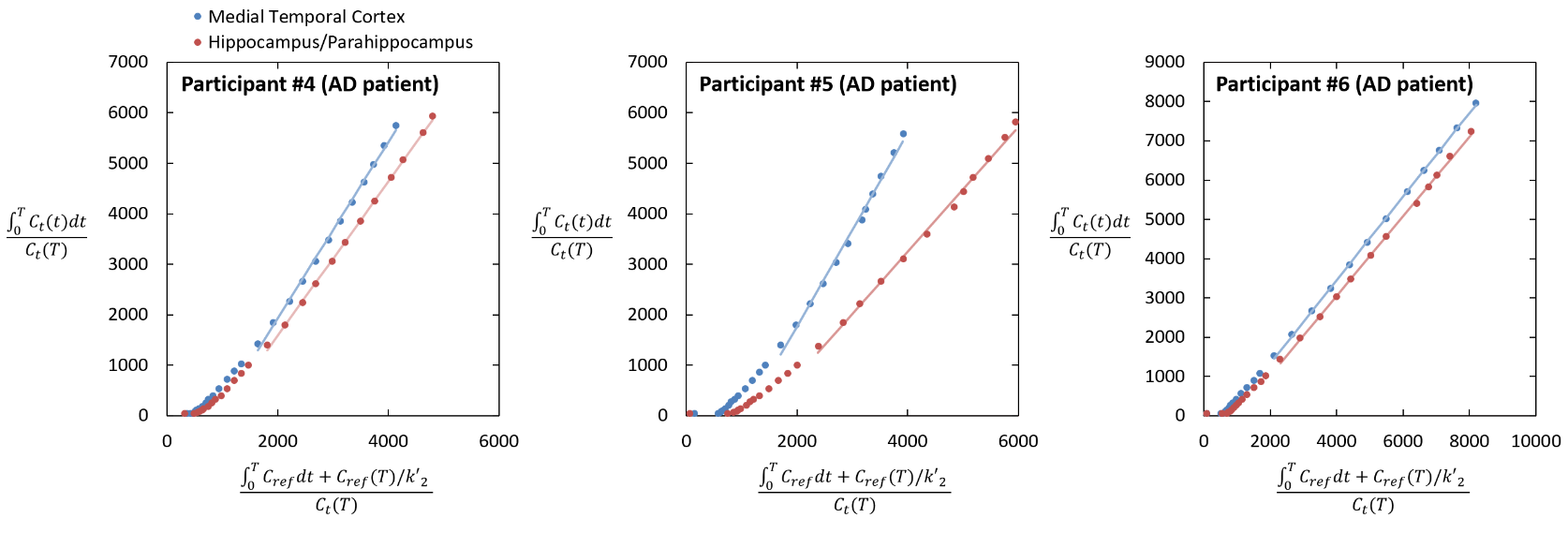


**Supplemental Table 1.** Radiation dosimetry estimates (OLINDA ver. 1.1) for [^18^F]MK-6240: Organ doses and effective dose estimated from three elderly healthy subjects.

| Target organs | Organ dose (µGy/MBq) |
| --- | --- |
| Adrenals | 11.3±0.3 |
| Brain | 18.5±7.2 |
| Breasts | 6.7±0.4 |
| Gallbladder wall | 240.7±71.4 |
| LLI wall | 47.1±18.7 |
| Small intestine | 45.5±20.7 |
| Stomach wall | 14.7±1.1 |
| ULI wall | 54.0±21.5 |
| Heart wall | 29.2±6.5 |
| Kidneys | 27.5±1.4 |
| Liver | 20.8±1.2 |
| Lungs | 38.1±15.4 |
| Muscle | 8.6±0.2 |
| Ovaries | 17.7±1.8 |
| Pancreas | 13.0±0.3 |
| Red marrow | 13.8±0.7 |
| Osteogenic cells | 13.5±0.6 |
| Skin | 5.7±0.1 |
| Spleen | 11.4±2.6 |
| Testes | 8.3±0.2 |
| Thymus | 8.3±0.5 |
| Thyroid | 6.8±0.1 |
| Urinary bladder wall | 124.7±11.2 |
| Uterus | 19.4±1.9 |
| Total body | 10.5±0.7 |
|  |  |
| Effective dose (µSv/MBq) | **26.4±1.2** |

Data are mean±SD
